# Identification of a novel mutation in the mechanoreceptor-encoding gene CXCR1 in patients with keloid

**DOI:** 10.1007/s00403-018-1847-3

**Published:** 2018-06-21

**Authors:** Qiguo Zhang, Liangqi Cai, Mian Wang, Xiaoping Ke, Xiaoyan Zhao, Yijin Huang

**Affiliations:** The Department of Dermatology, The First Affiliated Hospital of Xiamen University, Xiamen, 361003 China

**Keywords:** Mechanoreceptor, CXCR1, Keloid, Gene screening, Mutation

## Abstract

Keloids are skin fibroproliferative tumors characterized by locally invasive growth of fibroblasts and excessive collagen deposition. The objective of this study is to investigate the molecular basis of the keloid scar by studying the mutation of related genes. We performed gene screening of mechanoreceptors by quantitative polymerase chain reaction (qPCR), Sanger sequencing to detect the CXCR1gene mutation, and immuno-histochemistry to determine CXCR1 protein expression. Among the genes encoding mechanoreceptors, the expression of CXCR1 mRNA was significantly higher in keloid scar tissues than in the surrounding tissues of normal controls (*P* < 0.05). Sequencing analysis identified a novel missense mutation, c.574G > A (p.Gly192Glu). Immunohistochemistry showed heightened protein expression of CXCR1 in keloid scars as compared to controls. Our findings indicate that CXCR1 gene mutation and altered protein expression are associated with keloid scar development. Identification of the CXCR1 gene mutation might provide insights into the molecular mechanism underlying keloid scar and underscores the potential importance of mechanoreceptors in keloid scar pathogenesis.

**Electronic supplementary material:**

The online version of this article (10.1007/s00403-018-1847-3) contains supplementary material, which is available to authorized users.

## Introduction

Keloid is a dermal fibroproliferative tumor that forms at the site of a cutaneous injury and is characterized by heterogeneity, excessive collagen accumulation and locally aggressive invasion [9]. The excessive deposition of fibroblast-derived collagens I and III in the growing margin of keloid scars as compared to intralesional and extralesional sites [15] greatly concern patients physically, psychologically and cosmetically [1, 19]. The molecular mechanism underlying keloid pathogenesis remain poorly understood [10].

Genetic predisposition and the environmental and epigenetic factors collectively contribute to the formation of keloid [5]. Keloids at chest areas and the lower extremities are most vulnerable to recur even after completion of successful treatment [13], which implicates that high mechanical tension actively contributes to keloid. In addition, keloids tend to develop at high-tension sites such as the chest, back, and extremities. While some keloids grow at sites with low tension including the auricle and axilla, there is actually another type of mechanical tension that includes inflammatory tension at these sites. Ogawa et al. reported that keloid and hypertrophic scars in the reticular dermis result from chronic inflammation [12], which favors the possibility that a primary inflammatory lesion may facilitate the formation of keloid, which suggests that mechanical tension plays a potentially important role in the development of keloid.

In this study, we analyzed the transcriptional level of known mechanoreceptor genes, and employed Sanger sequencing to identify potential mutations in these genes. We identified the expression of a novel missense mutation, c.574G > A (p.Gly192Glu) in the CXCR1 gene. Immunohistochemical staining confirmed an elevated expression of CXCR1 at the protein level in keloid as compared to controls.

## Materials and methods

Informed written consent was obtained from all subjects (Table S1) in this study according to the tenets of the Declaration of Helsinki. This study was approved by the Institutional Review Board at the First Affiliated Hospital of Xiamen University.

### A retrospective survey on body site distribution of keloids

Information relevant to those patients enrolled in our department presenting with keloid was registered. This information included the patient’s name, gender, age, site of the keloids, and age of onset from Nov. 2015 to Nov. 2017. During this time, 315 patients were recorded, following which, we statistically analyzed the keloid sites.

### Patients’ Identification and DNA/RNA Isolation

Ten unrelated patients with keloid were diagnosed at the Department of Dermatology, the First Affiliated Hospital of Xiamen University (Xiamen, China). Two experienced dermatologists made an independent diagnosis based on the clinical findings. After obtaining informed consent, the patients were enrolled. Peripheral blood was drawn from four of the patients (i.e., P1–P4). Tissue samples were obtained by biopsy on six of the patients (i.e., P5–P10) and included only the keloid scar for gene screening by qPCR. To compare mechanoreceptor genes expression under different tensions, we collected specimens of post-operative healthy skin of patients with lipomyoma following plastic surgery at high-tension sites of the body (i.e., C1–C6) and post-operative foreskins after preputial circumcision following urinary surgery as the low-tension group (i.e., C7–C12).

Genomic DNA was purified from peripheral blood leukocytes using the DNA Isolation kit (CWBIO, Jiangshu, China) according to the manufacturer’s instructions. Total RNA of lesional skin tissues from six patients with keloid and unaffected skins from normal and apparently healthy controls at different tension sites was isolated using TRIzol (Invitrogen). One microgram of RNA was used to conduct reverse transcription with a Takara RT kit (6210A).

### Candidate-gene screening for keloid

Total RNA from six keloid patients (i.e., P5–P10) and 12 unaffected healthy controls (i.e., C1–C6 representing the middle tension group and C7–C12 representing the low tension group) was employed for candidate-gene screening of the keloid scar. Quantitative polymerase chain reaction (qPCR) was performed on an ABI 7500 thermocycler (Applied Biosystems, Foster City, CA, USA) using 1 µL of cDNA and SYBR Green real-time PCR Master Mix (Takara, China). GAPDH was used as a housekeeping gene for normalization as previously described [17]. The primers are listed in Table S2.

### PCR Amplification and Sanger sequencing

Genomic DNA from four keloid patients (P1–P4) was used for mutation analysis of the CXCR1 and CXCR2 genes, using described primers (Table S3) and conditions. PCR was carried out in a 25 µL total volume, containing 20 ng genomic DNA, 10 mM Tris–HCl (pH 8.3), 50 mM KCl, 3.0 mM MgCl_2_, 0.01% gelatine, 0.2 mM dNTPs, 10 pmol of each primer, and 0.75 U of Hot Stars Taq polymerase (QIAgene, Germany). The PCR program was set as described below: Hot Stars Taq activation at 95 °C for 10 min was followed by 35 cycles of PCR, each having a denaturation at 95 °C for 30 s, and an annealing temperature of 60 °C for 30 s and a primer extension temperature of 72 °C for 45 s, and a final extension at 72 °C for 5 min.

### Direct sequencing was performed with an ABI BigDye Terminator v3.1 Cycle

This procedure was done using a commercial Sequencing Kit (Applied Biosystems, California, USA) and analyzed on an ABI 3730 genetic analyzer (Applied Biosystems, USA). The new variants were then analyzed in 100 normal chromosomes to exclude the possibility of polymorphisms.

### Immunohistochemistry Imaging of CXCR1

The CXCR1 protein was assessed by immunohistochemistry (IHC). The keloid lesions and normal-appearing skin around the keloid from the same patients were fixed in 10% Tris-buffered formalin and subsequently paraffin embedded for analysis. Immunodetection was performed using a standard immunohistochemistry protocol, as described previously [2], with antibodies directed against CXCR1 (Table S4). Twenty-four images were obtained by microscopy. Immunohistochemical quantification and analysis were performed as described previously [14].

## Results

### Body distribution of keloid scar in the Chinese population

**Fig. 1:**
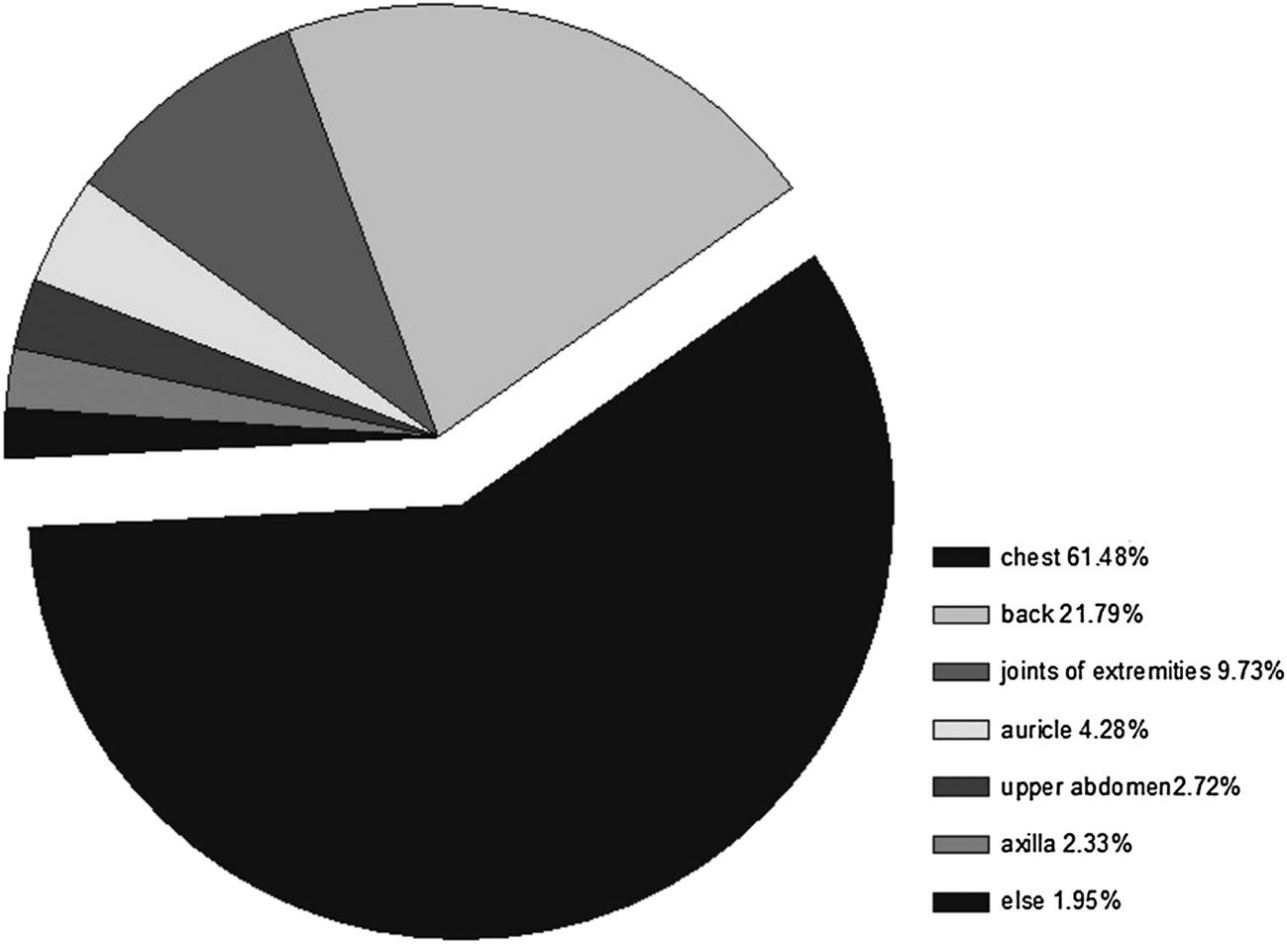
Regional distribution of keloid scar through the body. It was determined that 61.48% of keloid scars were located at the chest, 21.79% at the back, and 16.73% at other sites including joints of extremities, auricle, abdomen, axilla and buttock

In the Chinese population, keloid scar was mainly distributed at sites of high tension including the chest (61.48%) and the back (21.79%), and joints of the extremities (9.73%). Rarely were keloids seen at sites of low tension such as the auricle, upper abdomen and axilla (Fig. 1).

### Quantitative assay of the mechanoreceptors-encoding genes in keloid scars

**Fig. 2:**
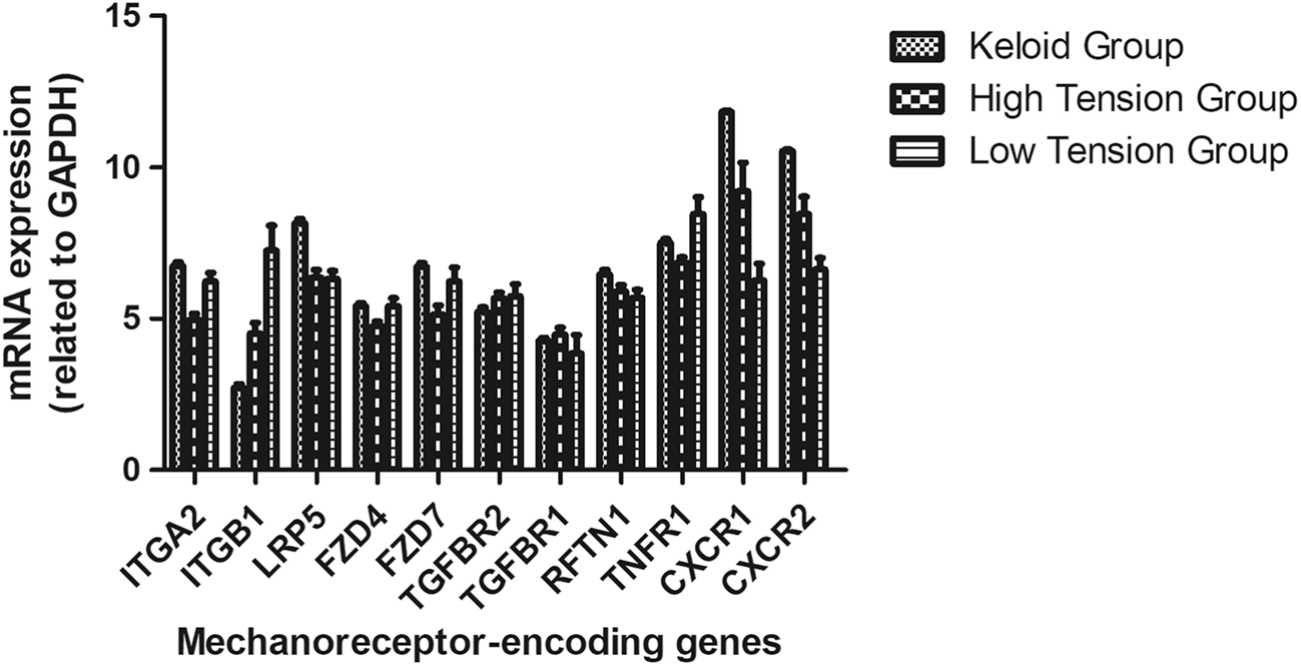
Quantitative analysis of mechanoreceptor-encoding genes in keloid scars. qPCR Determined the expression of mechanoreceptor-encoding genes in keloid scars and an elevated expression of the CXCR1 gene was found in keloid tissues as compared healthy skin tissues with high and low tension (*P* < 0.05). The elevated expression was also found in healthy skin tissues with high tension as compared low tension (*P* < 0.05)

Considering potential involvement of mechanical tension in the keloid, we analyzed the expression of some known mechanoreceptor-encoding genes, including CXCR1, CXCR2, TGFBR1, TGFBR2, ITGA2, ITGB1, LRP5, FZD4, FZD7, RFTN1, and TNFR1. We found significantly elevated CXCR1 expression in keloid tissues as compared to healthy control skin tissues with high and low tension (*P* < 0.05). We also found significantly elevated CXCR1 expression in healthy control skin tissues with high tension as compared to low tension (*P* < 0.05; Fig. 2).

### Expression analysis of CXCR1 in keloids by IHC

**Fig. 3:**
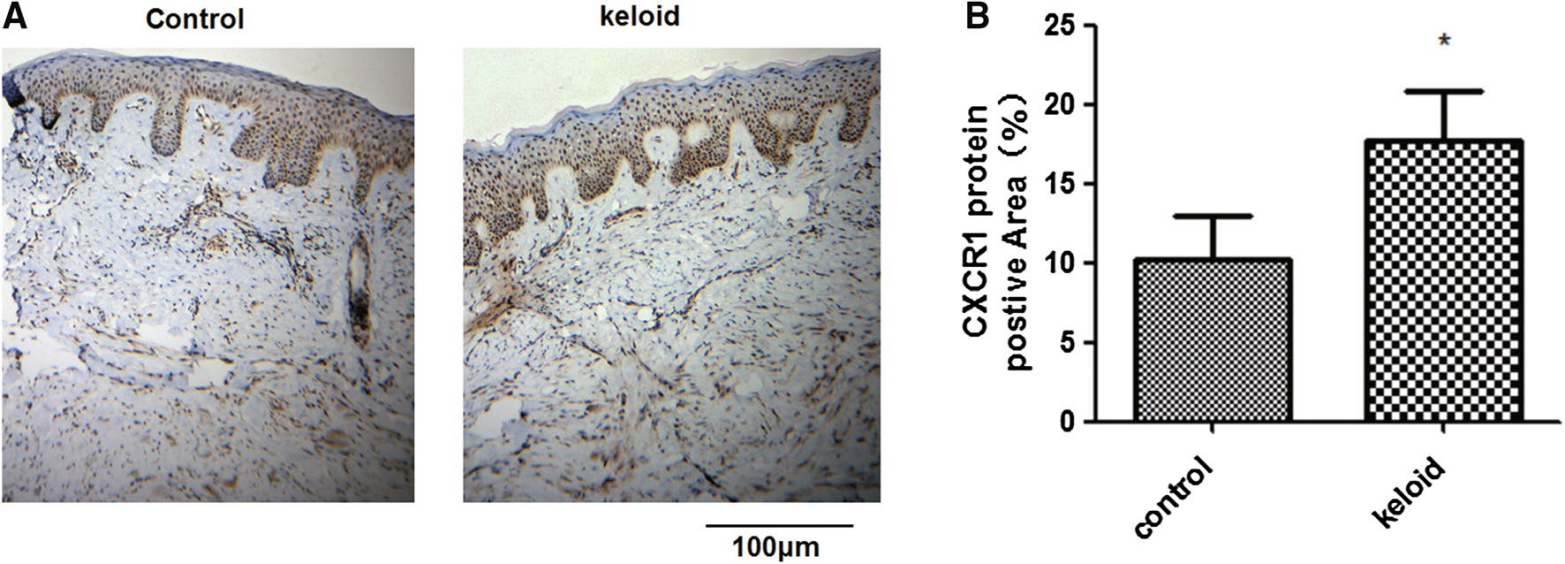
Measurement of CXCR1 protein expression in keloid tissues. Four pairs of tissue samples from keloid and surrounding regions were used to detect the protein levels of CXCR1 by immunohistochemistry. **a** Typical immunohistochemical images are shown. **b** Pooled data from **a**. The positive signaling with CXCR1 antibody staining was significantly stronger in keloid tissues than in the surrounding controls. The images shown are representative of four independent experiments. *P < 0.05

Immunohistochemical staining for CXCR1 was performed on keloids and surrounding normal controls that were biopsied from individuals with keloid. CXCR1 protein expression was significantly increased as compared with controls (Fig. 3a, b) (*P* < 0.05).

### Mutation analysis of CXCR1 gene

**Fig. 4:**
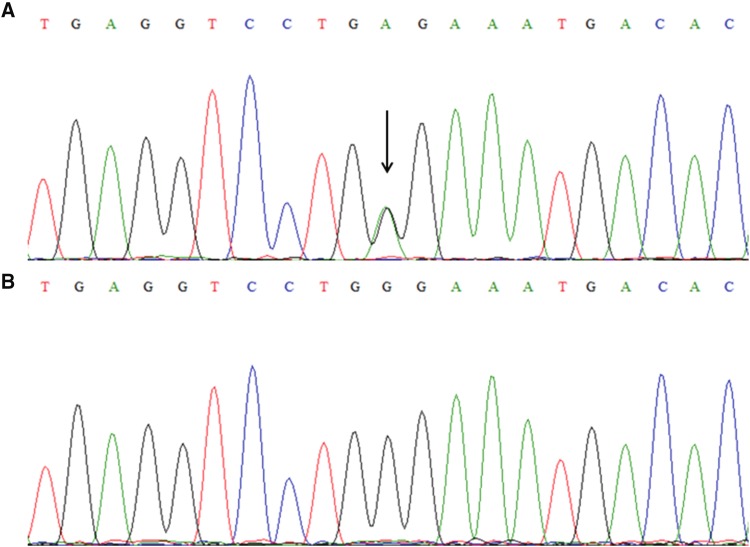
Sequence analysis in a sporadic case with keloid. **a** Direct sequencing identified a heterozygous c.574G > A (p.Gly192Glu) mutation in the CXCR1 gene of a sporadic case. **b** The wild-type allele was found in healthy controls

Sequence analysis identified a novel missense mutation, c.574G > A, which indicated a change of G to A at nucleotide position 574 in the CXCR1 gene, resulting in a substitution of glycine (GGG) to glutamate (GAG) at position 192 (p.Gly192Glu) in a sporadic case (Fig. 4). This mutation was not found in 100 unrelated individuals.

## Discussion

To our knowledge, two tension types are likely involved in keloid formation, including mechanical tension and inflammatory tension. For the first time, we propose the concept of “inflammatory tension” to interpret why keloids also occur at low mechanical tension sites such as the auricle and axilla after local purulent infection. Actually, the term of inflammatory tension in the manuscript was used to try to describe the intracellular tension by local inflammatory reaction, such as cellular swelling. Keloid scars tend to develop at high tension sites as reported herein. Another article reported a consistent result of a stretching tension that might represent a major mechanical force that drives keloid generation according to the site specificity of keloids. Keloids might also result from an excessive response or functional failure of either dermal cell mechanoreceptors or mechanosensitive nociceptors of sensory fibers in the skin. In other words, keloids are possible disorders of the mechano-receptor, mechanosensor or mechanosensitive nociceptor [11].

Some signaling pathways are thought to mediate keloid formation. Transforming growth factor-beta (TGF-beta)/Smad signaling plays a key role in excessive fibrosis and keloid formation [6] Wnt signaling also plays key roles in various cellular functions including proliferation, differentiation, survival, apoptosis and migration, [8] which can exacerbate keloid cell proliferation and inhibit keloid cell apoptosis through its interaction with telomerase [16].

We have analyzed some known mechanoreceptor genes that had been previously reported [3, 7] by qPCR. We found elevated protein expression of CXCR1 in keloid tissues as compared to healthy controls not only in the high tension group but also in the low tension group. Immuno-histochemical staining (IHS) identified differential CXCR1 protein expression in keloid tissues compared with controls. Unexpectedly, IHS showed a significantly higher expression of CXCR1 in the epidermis as compared with the dermis. Observations indicated that cells in the epidermis are also involved in keloid formation. We also found a novel heterozygous missense mutation c.574G > A(p.Gly192Glu) in the CXCR1 gene in a patient with keloid by Sanger sequencing. PolyPhen-2 suggested that the Gly192Glu mutation was “probably abnormal.”

CXCR1 is a G-protein-coupled chemokine receptor for its cognate ligand CXCL8. CXCR1 is essential not only for activation and trafficking of inflammatory mediators but also for tumor progression and metastasis [3, 7]. The CXCL8-CXCR1 signaling pathway is involved in the pathogenesis of several diseases including chronic obstructive pulmonary diseases (COPD), asthma, cystic fibrosis and cancer [4]. Furthermore, Zeng et al. reported that CXCR1 is a potential mechanosensor for mechano-transduction of hemodynamic forces [18]. These data suggest a potential role of CXCR1 in keloid formation. However, no study has yet been conducted to determine the correlation between CXCR1 and keloid formation. We demonstrated heightened CXCR1 expression in keloid scars as compared to the surrounding normal skin and a novel missense mutation in the CXCR1 gene, which indicated the importance of the CXCR1 gene in mediating keloid formation.

Taken together, our findings suggest that CXCR1 probably participates in human keloid formation as a mechano-receptor. To formally demonstrate this hypothesis, further functional studies are required.

## Electronic supplementary material

Below is the link to the electronic supplementary material.

Supplementary material 1 (PDF 117 KB)
