# Supplementary material for: Identification of a novel mutation in the mechanoreceptor-encoding gene CXCR1 in patients with keloid
Source: Arch Dermatol Res. 2018 Jun 21;310(7):561–6. doi: 10.1007/s00403-018-1847-3 (PMC6096561; doi:10.1007/s00403-018-1847-3)
Supplement: Supplementary file 1 — Supplementary material 1 (PDF 117 KB) [file 403_2018_1847_MOESM1_ESM.pdf]

Table S1. Basic information of subjects

Subjects	Gender	Age (Years old)	Location	Samples	Isolation	Application	Tension
patient 1	male	25	chest	peripheral blood	DNA	mutation analysis	–
patient 2	male	35	chest	peripheral blood	DNA	mutation analysis	–
patient 3	female	29	chest	peripheral blood	DNA	mutation analysis	–
patient 4	female	18	back	peripheral blood	DNA	mutation analysis	–
patient 5	male	19	chest	skin tissue	RNA	gene screening by qPCR	high
patient 6	female	26	chest	skin tissue	RNA	gene screening by qPCR	high
patient 7	male	34	chest	skin tissue	RNA	gene screening by qPCR	high
patient 8	female	29	back	skin tissue	RNA	gene screening by qPCR	high
patient 9	male	33	back	skin tissue	RNA	gene screening by qPCR	high
patient 10	female	41	back	skin tissue	RNA	gene screening by qPCR	high
patient 11	male	19	chest	skin tissue	RNA	RNA-seq	–
patient 12	male	34	back	skin tissue	RNA	RNA-seq	–
patient 13	female	35	auricle	skin tissue	RNA	RNA-seq	–
control 1	female	17	scalp	skin tissue	RNA	gene screening by qPCR	middle
control 2	male	28	arm	skin tissue	RNA	gene screening by qPCR	middle
control 3	female	25	arm	skin tissue	RNA	gene screening by qPCR	middle
control 4	female	16	arm	skin tissue	RNA	gene screening by qPCR	middle
control 5	male	34	neck	skin tissue	RNA	gene screening by qPCR	middle
control 6	female	29	lower leg	skin tissue	RNA	gene screening by qPCR	middle
control 7	male	8	prepuce	skin tissue	RNA	gene screening by qPCR	low
control 8	male	11	prepuce	skin tissue	RNA	gene screening by qPCR	low
control 9	male	13	prepuce	skin tissue	RNA	gene screening by qPCR	low
control 10	male	9	prepuce	skin tissue	RNA	gene screening by qPCR	low
control 11	male	18	prepuce	skin tissue	RNA	gene screening by qPCR	low
control 12	male	21	prepuce	skin tissue	RNA	gene screening by qPCR	low

Article title: Identification of a novel mutation in the mechanoreceptor-encoding gene CXCR1 in patients with keloid

Journal name: Archives of Dermatological Research

Author names: Qiguo Zhang, Liangqi Cai, Mian Wang, Xiaoping Ke, Xiaoyan Zhao, Yijin Huang

Affiliation: The First Affiliated Hospital of Xiamen University

e-mail address of the corresponding author: zqgdreamer@163.com; h8883697@126.com

Table S2. List of primers used for qPCR screening of RNAseq data of select genes

Gene	Forward/Reverse	Sequence	Amplicon Size (bp)	Primer Bank ID
ITGA2	forward	CCTACAATGTTGGTCTCCCAGA	106	116295257c1
	reverse	AGTAACCAGTTGCCTTTTGGATT		
ITGB1	forward	CCTACTTCTGCACGATGTGATG	128	182507160c1
	reverse	CCTTTGCTACGGTTGGTTACATT		
LRP5	forward	ACTCGCTGTGAGGAGGACAAT	82	119709831c1
	reverse	GGCAGGCGCATGTGTAGAA		
FZD4	forward	CCTCGGCTACAACGTGACC	155	333609263c1
	reverse	TGCACATTGGCACATAAACAGA		
FZD7	forward	GTGCCAACGGCCTGATGTA	111	4503832c1
	reverse	AGGTGAGAACGGTAAAGAGCG		
TGFBRII	forward	GTAGCTCTGATGAGTGCAATGAC	132	133908633c1
	reverse	CAGATATGGCAACTCCCAGTG		
CXCR1	forward	CTGACCCAGAAGCGTCACTTG	139	29171679c1
	reverse	CCAGGACCTCATAGCAAACTG		
CXCR2	forward	CCTGTCTTACTTTTCCGAAGGAC	82	269973857c1
	reverse	TTGCTGTATTGTTGCCCATGT		
TGFBRI	forward	ACGGCGTTACAGTGTTTCTG	167	195963411c1
	reverse	GCACATACAAACGGCCTATCTC		
RFTN1	forward	ATGGGTTGCGGATTGAACAAG	118	41872576c1
	reverse	AGCGGTATTCATAGGACACATCT		
TNFR1	forward	TGCTGTACCAAGTGCCACAA	80	self-designed
	reverse	CACTCCCTGCAGTCCGTATC		
FGF5	forward	CACTGATAGGAACCCTAGAGGC	196	73486656c1
	reverse	CAGATGGAAACCGATGCCC		

Article title: Identification of a novel mutation in the mechanoreceptor-encoding gene CXCR1 in patients with keloid

Journal name: Archives of Dermatological Research

Author names: Qiguo Zhang, Liangqi Cai, Mian Wang, Xiaoping Ke, Xiaoyan Zhao, Yijin Huang

Affiliation: The First Affiliated Hospital of Xiamen University

e-mail address of the corresponding author: zqgdreamer@163.com; h8883697@126.com

Table S3. List of primers used for gene mutation analysis

Primer name	Primer sequence (5'-3')
CXCR1-a-F	TGAATCCGAGCTACTAAATCACA
CXCR1-a-R	AAAATCCAGCCATTCACCTTG
CXCR1-b-F	GCCTTGGCCGACCTACTCTT
CXCR1-b-R	CGAAAATTTTGGCCGATGAA
CXCR1-c-F	CGCTGTTTGTCATGCTGTTC
CXCR1-c-R	CCTTCCACACACAACCTCAG
CXCR2-a-F	CCTTCATCTCTCTTCTATAGGTCAGG
CXCR2-a-R	ACAGGAATGTGCCAAAAATC
CXCR2-b-F	CGACCTACTCTTTGCCCTG
CXCR2-b-R	TGAGGACGACAGCAAAGATG
CXCR2-c-F	CCTGCGTACGCTGTTTAAGG
CXCR2-c-R	GGACATGAGGCTTGGAATGT

Article title: Identification of a novel mutation in the mechanoreceptor-encoding gene CXCR1 in patients with keloid

Journal name: Archives of Dermatological Research

Author names: Qiguo Zhang, Liangqi Cai, Mian Wang, Xiaoping Ke, Xiaoyan Zhao, Yijin Huang

Affiliation: The First Affiliated Hospital of Xiamen University

e-mail address of the corresponding author: zqgdreamer@163.com; h8883697@126.com

Table S4. Description of antibodies used for IHC imaging of CXCR1

Protein	Antibody	Vendor	Cat#	Dilution
CXCR1	rabbit polyclonal IgG	Abcam	ab85103	1:100

Article title: Identification of a novel mutation in the mechanoreceptor-encoding gene CXCR1 in patients with keloid

Journal name: Archives of Dermatological Research

Author names: Qiguo Zhang, Liangqi Cai, Mian Wang, Xiaoping Ke, Xiaoyan Zhao, Yijin Huang

Affiliation: The First Affiliated Hospital of Xiamen University

e-mail address of the corresponding author: zqgdreamer@163.com; h8883697@126.com
